# Oculomotor Evidence for Top-Down Control following the Initial Saccade

**DOI:** 10.1371/journal.pone.0023552

**Published:** 2011-09-08

**Authors:** Alisha Siebold, Wieske van Zoest, Mieke Donk

**Affiliations:** Department of Cognitive Psychology, Vrije Universiteit Amsterdam, Amsterdam, The Netherlands; Monash University, Australia

## Abstract

The goal of the current study was to investigate how salience-driven and goal-driven processes unfold during visual search over multiple eye movements. Eye movements were recorded while observers searched for a target, which was located on (Experiment 1) or defined as (Experiment 2) a specific orientation singleton. This singleton could either be the most, medium, or least salient element in the display. Results were analyzed as a function of response time separately for initial and second eye movements. Irrespective of the search task, initial saccades elicited shortly after the onset of the search display were primarily salience-driven whereas initial saccades elicited after approximately 250 ms were completely unaffected by salience. Initial saccades were increasingly guided in line with task requirements with increasing response times. Second saccades were completely unaffected by salience and were consistently goal-driven, irrespective of response time. These results suggest that stimulus-salience affects the visual system only briefly after a visual image enters the brain and has no effect thereafter.

## Introduction

Imagine you are looking for your friend in a large shopping mall crowded with people. Even though you know exactly what your friend looks like, you have difficulty identifying him in the turmoil because your gaze is automatically captured by other people and the colorful and brightly blinking advertisements of the shops. In the literature on overt visual selection the previous situation is, in one way or another, a commonly cited example of how salient objects capture one's eyes automatically, thereby hindering or slowing goal-directed visual search. However, is it really true that salient objects attract our gaze automatically? Even if this is the case, are we really distracted by salient events when we scrutinize our visual environment or might the impact of the effect be negligible?

These questions have been investigated for multiple decades without definitive results. On the one hand, empirical evidence does indeed show that salient objects or features in the visual field receive selective priority by attracting attention and the eyes [1]–[7]. However, this evidence is primarily derived from reaction time (RT) studies [8]–[13] and studies in which the results are based on the analysis of participants' initial eye movements only [14]–[20]. Studies examining overt visual selection behavior under free-viewing conditions, i.e. those in which multiple eye movements are made, do not provide unequivocal evidence for the idea that visual selection is salience-driven [21]–[34].

For instance, Parkhurst, Law, & Niebur (2002) examined the relationship between stimulus salience and observers' fixation locations of free-viewing static images displaying complex artificial and natural scenes. They obtained a significant correlation between fixation locations and stimulus salience, albeit this correlation became weaker over time, i.e. over multiple eye movements. This suggests that selection is consistently salience-driven over multiple eye movements. In contrast, a number of other studies have demonstrated that eye movement behavior under free-viewing conditions is unaffected by salience and primarily under goal-driven control [2]–[34]. For instance, Underwood et al. (2006) recorded eye movements while observers searched for a target in pictures of natural office scenes containing two objects differing in relative salience. The results indicated that the presence of the high saliency object was ineffective in distracting observers from selecting the less salient target object, suggesting that specific task requirements can provide a “[…] cognitive override that renders saliency secondary.”

These inconclusive and contradictory results regarding the contribution of salience-driven and goal-driven control in visual selection are manifested in a continuing debate that is far from being settled. A definitive conclusion is hampered by widely differing approaches (e.g., RT versus eye movement studies; free-viewing versus single eye movements, static versus dynamic scenes etc.) rendering any direct comparisons between studies difficult if not impossible. One factor, however, that might be crucial in determining whether or not visual selection is salience-driven or goal-driven, is time. It has been shown that the contribution of salience-driven and goal-driven processes is contingent upon the timing of an individual saccade relative to the presentation of a visual display [14]–[20]. For instance, Donk and van Zoest (2008) instructed participants to make one single eye movement to the most salient element in a search display amongst a distractor and multiple homogeneously aligned background elements. They investigated how the proportion of correct eye movements varied as a function of saccadic latency and found that eye movements were accurate for very brief saccadic latencies but dropped to chance level when latencies were longer. They concluded that salience-driven processes do affect visual selection but only during a brief period after the presentation of a display. Hunt, Von Muehlenen, and Kingstone (2007) drew a similar conclusion. In their study, observers had to make an eye movement to a color singleton in the presence of an irrelevant distractor. Eye movements were registered and the proportion of trials in which the eyes were erroneously captured by the onset distractor was examined separately for different quartiles of the saccadic latency distribution. The results showed that short-latency responses were often misdirected towards the distractor whereas long-latency responses were not. Finally, van Zoest and Donk (2008) investigated the time-course of goal-driven control within an initial eye movement during visual search. They instructed participants to make one eye movement to a prespecified target, which differed in stimulus-salience and/or the feature dimension from simultaneously presented non-targets and one distractor. Performance accuracy in selecting the target was investigated as a function of saccadic latency and the results indicated that goal-driven processes increased as a function of response latency. Together, these results indicate that the timing of a response within an initial eye movement is crucial in determining the contribution of both salience-driven and goal-driven processes to overt visual selection.

If response time is essential in determining how a single eye movement is controlled, it is important to determine whether the mode of control is also time-dependent in a *sequence* of eye movements. In other words, is salience-driven and goal-driven control contingent upon the response timing of each individual saccade in a sequence of multiple saccades? Surprisingly, not much is known about the temporal characteristics of selective control over multiple eye movements.

The present study investigated in two different experimental tasks how the second eye movement in a sequence is affected by salience-driven and goal-driven processes, respectively, while taking into account the response time of each individual eye movement. In line with previous studies, response time of the initial saccade refers to the time interval between the onset of the search display and the initiation of a saccade, i.e. saccadic latency. Given the nature of the paradigm, in which static stimuli were presented, second saccades were not directly triggered by a stimulus onset and therefore response time could not be expressed in terms of saccadic latency, However, research has shown that the intersaccadic interval (ISI), the time interval between the start of fixation of the previous saccade and the initiation of the following saccade, can be regarded as an equivalent measure of saccadic latency [35]. Therefore, the response time of the second saccade refers to ISI. Participants were instructed to search for a small probe dot superimposed upon one of three differently salient singletons (Experiment 1) or to search for the only right-tilted singleton (Experiment 2) in the display. Eye movements were recorded and categorized as being directed to either the most, medium, or least salient singleton, separately for initial and second eye movements per quintile of the respective response time distribution.

For a complete account of visual selection, it is necessary to integrate both findings of salience-driven and goal-driven processes in one framework. One way of doing this is by assuming varying time-courses of the relative contributions of both processes. Based on previous findings [14], [15], [18] two potential patterns of time-courses come into consideration: Assuming a *saccade-confined time-course view*, the relative contribution of salience-driven and goal-driven processes is dependent on the response time of each individual saccade in a *sequence* of eye movements. For *each* eye movement, visual selection is salience-driven only for very fast responses. As time elapses, stimulus-salience becomes irrelevant and visual selection becomes increasingly goal-driven. Importantly in this view, the pattern of salience-driven and goal-driven processes is identical over multiple saccades, with salience-driven processes being reinstated after every fixation for each following saccade anew. Alternatively, it is possible that the relative contribution of salience-driven and goal-driven processes is dependent on the response time of the *initial eye movement only*. Only within the initial saccade is visual selection salience-driven for very fast responses. Subsequently, saccadic selection is purely goal-driven, not only for slower responses but for all following eye movements irrespective of response time. According to this *absolute time-course view*, stimulus-salience plays only a very limited role in guiding visual selection.

## Experiment 1

The aim of Experiment 1 was to investigate how stimulus-salience affects visual search over a sequence of eye movements. To this end, participants were instructed to search for a very small black probe dot superimposed upon one of three differently orientated singletons relative to multiple uniformly aligned background lines. On each trial, the singleton that contained the target dot could be the most, medium, or least salient singleton in the display. On two-thirds of the trials no target was present. The probe dot could only be identified with foveal vision so that participants were forced to make multiple eye movements in order to determine whether the target was present. The relative salience of the singletons was irrelevant to the task so that task-requirements and subsequent target selection were independent of salience information. Eye movements were recorded and initial and second saccades were separately analyzed as a function of response time. In line with the saccade-confined time-course view, it was expected that salience is reinstated after the initial eye movement. The proportion of second eye movements directed to any of the three singletons was predicted to vary with response time: salience effects were expected to be found only for fast-response saccades but not for slow response saccades. Alternatively, following the assumptions of the absolute time-course view, salience information is only transiently effective in the visual system. Accordingly, it was predicted that second eye movements are completely unaffected by salience information, irrespective of response time.

### Method

#### Participants

The sample in Experiment 1 consisted of 12 participants, who were either paid volunteers or psychology students at the Vrije University of Amsterdam. Ages ranged from 18 to 26 years (mean: 20.75 years); 11 of the participants were female. All participants reported normal or corrected-to-normal stereoscopic visual abilities. They were naïve with regard to the experimental stimuli and the purpose of the study. The experimental session lasted for approximately 60 minutes. Due to an excess of saccade destination errors (72.78%), the data of one participant was excluded from further analysis.

#### Ethics Statement

The present study, including the consent procedure, was approved by the ethics board of the Faculty of Psychology and Education (VCWE) and conducted according to the principles of the Declaration of Helsinki. Participants received information about the study and their rights and gave informed consent. As the study was not associated with any risks (non-invasive) for participants and all data obtained during this study were analyzed anonymously, only verbal consent was obtained.

#### Apparatus

A standard Pentium IV class computer with a processor speed of 2.3 GHz running C++ software package controlled stimulus presentation, timing of events, and acquisition of necessary response data. Stimuli were presented at eye-level, 75 cm from the chinrest, on a 21 Inch Iiyama SVGA (Super Video Graphics Array) monitor, running at 1024 by 768 pixel resolution, and refreshing at a rate of 100 Hz. Manual input was given through a standard keyboard placed on the table directly in front of the participant.

The position of the right eye was recorded every 2 ms by means of a head mounted video-based Eyelink II eye tracker (SR Research Ltd., Mississauga, Ontario, Canada), with a 500-Hz temporal resolution, a 0.01° of visual angle spatial resolution (noise limited), and a gaze position accuracy of 0.5°. Calibration of participants' eye movements was achieved with a grid of nine calibration points [36] in order to minimize errors resulting from non-linearity due to infrared source reflections. In the event of occasional excessive head or extreme eye movements during a block of trials, manual adjustment of drift corrections or complete recalibration was required.

Participants were tested individually in a dimly-lit, sound-attenuated research laboratory room, while the researcher monitored eye movement performance and supervised calibrations from a computer screen situated in an adjacent room.

#### Stimuli

The visual stimuli in Experiment 1 consisted of multiple white line segments (78.6 cd/m^2^) presented on a black background screen (0 cd/m^2^). Each line segment had a size of 0.76 * 0.15 cm and was presented in a 17 * 17 items square matrix grid with a raster width and height of 17.4 * 17.4 deg of visual angle. Three of these line segments, the singletons, differed in the orientation contrast relative to multiple homogeneously aligned background lines. Depending on the size of the orientation contrast, the singletons were referred to as the least (22.5°), medium (45°), and most salient singleton (67.5°), respectively. The singletons were randomly oriented to the left or right and were presented at a retinal eccentricity of 5.3 deg at central fixation. The group of uniformly oriented background elements was horizontally aligned. The target stimulus consisted of a black pixel randomly located at the center of one of the three singletons. The singletons were randomly presented at one of six potential grid locations on an imaginary circle around the center of the grid. Moreover, the presentation locations of the three singletons were subject to configurational constraints, in such a way that the positions of the singletons represented the intersections of one of two isosceles triangles on the imaginary circle. The central fixation preceding a trial, as well as the calibration stimuli consisted of a white disk of 0.3 cm in diameter.

#### Design and Procedure

Participants were seated in front of a computer screen with their forehead and chin resting on a head rest. Before the testing session, eye-movements were calibrated to a precision of 0.5 deg of visual angle. Participants were instructed to search for the probe dot and to press the spacebar if it was present.

**Figure 1:**
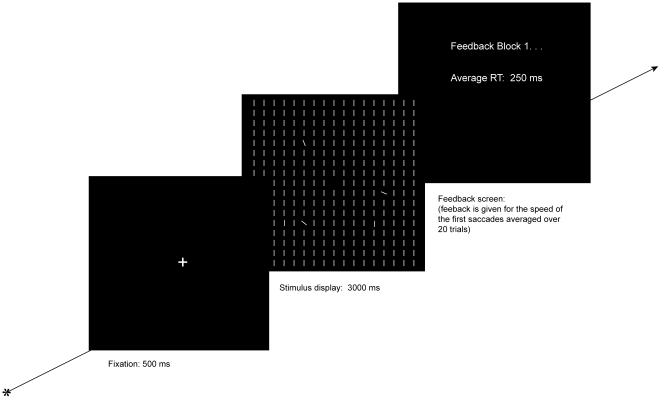
A typical trial sequence in Experiment 1. Prior to each trial, participants maintained fixated on a centrally presented disk until a stable fixation was detected. Upon depression of the spacebar, a drift correction was applied and a trial was initiated with the presentation of a centrally presented fixation cross. Following 500 ms, the fixation cross was replaced by the search display, which was presented for 3000 ms. Following each block, feedback regarding the speed of participants' initial saccade was provided and participants were given the opportunity to take a short break.

On two-thirds of the trials no probe dot was presented. The singleton locations within a particular configuration were mixed across trials. For an illustration of a typical trial sequence see Figure 1.

Prior to the main experimental testing session, participants were presented with 3 practice blocks of 20 trials each, identical to the experimental trials in order to familiarize them with the eye-tracking device and the experimental stimuli. These practice trials were not included in any subsequent analysis. Participants completed a total of 540 experimental trials presented in random order, equally distributed across 27 blocks. Eye-movements were recalibrated twice during the experimental session upon completion of every 180 trials. Following the testing session, participants were fully debriefed as to the purpose of the experiment.

A within-subject design was used with the factors Salience (most, medium, and least) and Response Time Bin (1, 2, 3, 4, and 5).

#### Data analysis

Fixation locations and durations of fixations and saccades were extracted from the raw eye tracking data by applying velocity, angle and duration criteria [36]. A trial was discarded if the response time of the initial eye movement remained below an arbitrary threshold of 80 ms (anticipation error) or exceeded an arbitrary threshold of 600 ms. An individual saccade was discarded if it fell outside the range of 3 deg of one of the three singletons. The complete dataset of a participant was excluded if more than 15% of trials had to be discarded. Only those trials were analyzed in which no probe dot was present. The reason for this was that the key press that was required to signal target presence may have potentially interfered with saccade programming or execution. For instance, participants might have moved their eyes to the keyboard during a manual response.

For initial saccades, a repeated measures analysis of variance (ANOVA) was performed on the proportion of eye-movements directed towards each of the three singletons with Salience (most, medium, and least) and Response Time Bin (1–5) as independent within-subject factors. In addition, pair-wise post-hoc comparisons were performed between each combination of levels within the two factors Salience and Response Time Bin.

Similar analyses were performed for second saccades, with the exception that separate ANOVAs were performed, contingent upon the landing position of the initial saccade.

### Results

Due to nonconformity to the previously established threshold criteria, 7.8% of initial saccades were excluded from analysis (3.6% due to an anticipation error, 0.3% due to the latency exceeding the threshold of 600 ms and 3.9% of initial saccades landed outside the range of 3 deg of visual angle of any of the three singletons.

#### Salience-driven influences on initial saccades

In order to compare the salience conditions across different points of the response time distribution, five bins were created. To this end, the overall distribution of each participant's response times of the initial saccades was rank ordered from fastest to slowest responses, irrespective of the saccade destination, and subsequently partitioned into five response time bins. For each participant, the proportion of initial saccades directed toward each of the three singletons was determined separately per bin and subsequently averaged across the sample in order to obtain the mean proportion of saccades directed toward each singleton per bin. A similar procedure was followed for the classification of second saccades, with the exception that the gaze proportions of only two singletons were examined per bin.

**Figure 2:**
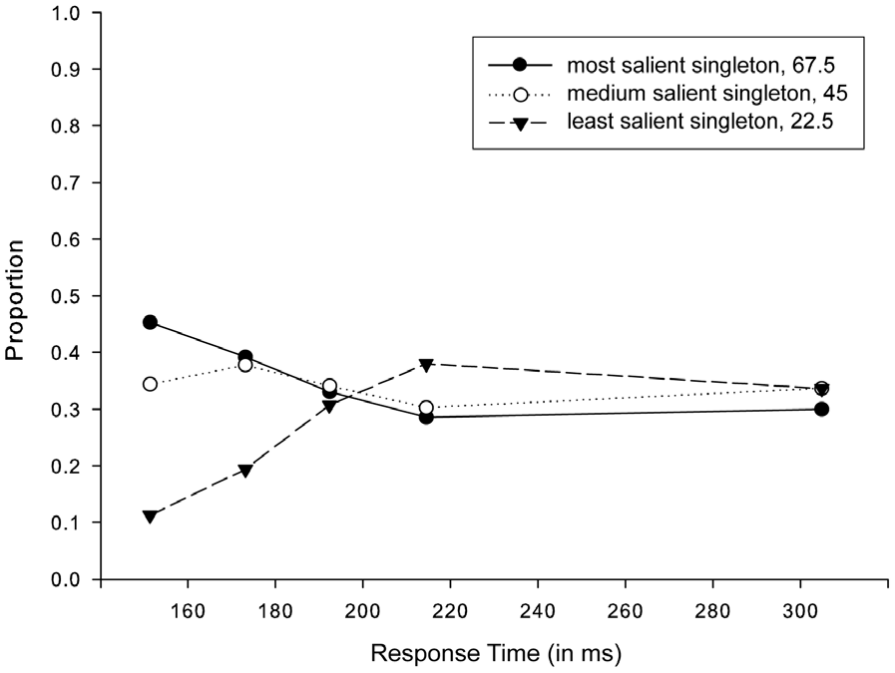
Salience-driven selection in initial saccades in Experiment 1. Proportions of initial saccades directed towards each of the three singletons (22.5°, 45°and 67.5°), separately for each bin of the response time distribution and irrespective of target identity.

The results of the ANOVA (see Figure 2) displayed a statistically significant main effect of Salience F(2, 20) = 22.196, MSE = .007, η^2^ = .155, p<.001]. Moreover, this effect was qualified by a significant interaction between Salience and Response Time Bin [F(8, 80) = 13.510, MSE = .008, η^2 = ^.445, p<.001], indicating that the proportion of initial eye movements directed towards each of the three singletons varied as a function of response time. Note, that as the proportions of eye movements directed to the three singletons add up to 1 for each bin, no first-order effects could be obtained for the factor Response Time Bin. Similarly, this applies to all subsequent analyses.

Post-hoc pair-wise comparisons between Salience separately for fastest and slowest responses revealed that, for fast-response saccades, participants were more likely to select the most salient singleton over the medium [t(10) = 3.074, p<.05] and the least salient singleton [t(10) = 9.350, p<.001] and more likely to select the medium salient singleton over the least salient singleton [t(10) = 6.943, p<.001]. This preference disappeared for slow-response saccades, with participants being equally likely to make an eye movement to either of the three differently salient singletons (most versus medium salient: t(10)<1; most versus least salient: t(10)<1; and medium versus least salient: t(10) = -1.357, p = .205).

#### Salience-driven influences on second saccades

For the analysis of second saccades only those trials were included in which the second eye movement landed on either of the two remaining singletons. Furthermore, in order to obtain reliable results, the data of two observers were excluded from the condition in which the initial eye movement landed on the least salient singleton as they contributed less than ten trials per bin to this salience condition.

**Figure 3:**
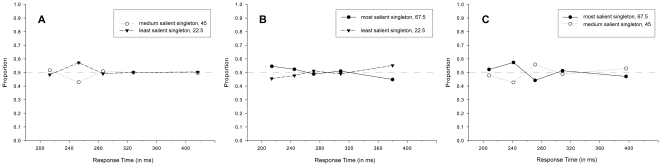
Salience-driven selection in second saccades in Experiment 1. Proportions of second saccades directed toward either of the two remaining singletons, separately for each bin of the response time distribution and irrespective of target identity, given that the initial saccade landed on A) the most, B) the medium, or C) the least salient singleton.

In order to investigate how oculomotor performance was affected by salience following the initial eye movement, the proportions of second saccades were analyzed as a function of response time, contingent upon the landing position of the initial saccade. Three separate repeated measures ANOVAs (given the initial saccades landed on A) the most, B) the medium, or C) the least salient singleton) were performed on the proportions of second saccades directed toward either of the two remaining singletons with Salience (2) and Response Time Bin (5) as within-subject factors. The results of all three analyses (see Figure 3) revealed neither a significant main effect of Salience [F<1, n.s. for all analyses] nor a significant interaction between Salience and Response Time Bin [for A): F(4, 40) = 1.402, MSE = .020, η^2^ = .090, p = .251, n.s.; for B): F<1, n.s.; and for C): [F(4, 32) = 2.129, MSE = .021, η^2 = ^.171, p = .100]. Irrespective of the landing position of the initial saccades, participants were equally likely to make a second eye movement toward either of the two remaining singletons. Furthermore, saccade destinations were invariant over response time. This indicates that participants displayed a consistent pattern of oculomotor performance for second saccades across all five bins.

An analysis of the data including probe dot trials showed the same pattern of results as reported above, both for initial and second saccades, indicating that the presence of the probe dot and the associated key press did not affect the results.

### Discussion

Regarding oculomotor performance of the initial saccades, the results of Experiment 1 indicated a change in the distribution of saccades directed to the three singletons with response time. In fact, the results remarkably resemble previous findings reported by Donk and van Zoest (2008). In line with their findings, saccades elicited shortly after the onset of the search display were primarily salience-driven, whereas saccades that were elicited later in time, after approximately 250 ms, were unaffected by salience.

Analyses of the second saccades showed that the pattern of oculomotor performance fundamentally differed from that of the initial saccades, in line with an absolute time-course view. No difference in performance was observed for saccades elicited early in time compared to those elicited later in time. Thus, irrespective of response time, participants were equally likely to make an eye movement to either of the two remaining singletons, even though they differed in relative salience. Importantly, this was true irrespective of whether the initial eye movement landed on the most, medium or least salient singleton. In all cases, participant's performance was at chance level, suggesting that salience-driven processes did not affect visual selection in any way.

Even though the pattern of results concerning initial saccades is remarkably similar to that reported previously [14], it is important to note that the nature of the search task used in Experiment 1 was different from the one used in previous work. In prior studies on saccadic target selection, observers were instructed to search for a specific target identity (e.g., the left-tilted among right-tilted elements) or for a certain salience level (e.g., the most salient singleton in the display). In the present study observers searched for a small probe dot superimposed upon one of the three singletons. In two-thirds of the trials no probe dot was presented, having urged observers to sequentially fixate each of the three singletons. One may argue that the absence of an effect of stimulus salience in the second eye movement is an artifact of the specific task used. Because of the small size of the probe dot, observers were forced to employ a very narrow focus or attentional window [37], [38]. This may have prevented potential effects of stimulus salience on performance, especially during the second eye movements when the attentional window might have been narrowly focused. Furthermore, salience effects have previously been found primarily for fast-response saccades. Potential salience effects might not have been represented in the data due to the relatively slow responses of second saccades.

In order to allow for a more direct comparison with previous work and to provoke faster second saccades, we designed a second experiment in which observers were instructed to search for a specific target identity. In contrast to Experiment 1, this should speed up responses, thereby increasing the probability of finding salience-driven effects during the second eye movement. In addition, the nature of this task allowed for an investigation of the time-course of goal-driven processes over multiple eye movements, rendering possible a direct comparison between the contribution of salience-driven and goal-driven processes within one experiment.

## Experiment 2

Experiment 2 was similar to Experiment 1 with the exception that instead of a visual search for a probe dot, observers were instructed to make an eye movement to the only right-tilted singleton in the search display. In contrast to the probe dot in Experiment 1, this target could be perceived with parafoveal vision. The right-tilted orientation singleton could be the most, the medium, or the least salient singleton in the display. As in Experiment 1, eye movements were recorded and initial and second saccades were separately analyzed as a function of response time. Relative singleton salience was completely irrelevant to the task, as was the case in Experiment 1.

Based on the findings of Experiment 1, it was predicted that the pattern of salience-driven processes is in accord with the absolute time-course view, which assumes that visual search is unaffected by stimulus-salience following a brief time-interval after stimulus presentation. Assuming that the time-course of goal-driven processes follows a complementary pattern to the time-course of salience-driven processes, it was furthermore predicted that visual search during initial saccades is unaffected by goal-driven processes shortly after stimulus onset but is increasing with increasing response time [18]. Following the absolute time-course view, visual search was expected to be consistently goal-driven, in line with the task requirements, irrespective of response time of second saccades.

### Method

#### Participants

A new sample was drawn for Experiment 2, consisting of 12 participants, who were either volunteers or psychology students at the Vrije University of Amsterdam. Ages ranged from 18 to 33 years (mean: 23.25 years); 10 of the participants were female. All participants reported normal or corrected-to-normal stereoscopic visual abilities. As in Experiment 1, participants were naïve to the experimental stimuli and the purpose of the study. The experimental session lasted for approximately 120 minutes. Participants received either course credits or 18,- € in return for taking part in the study.

#### Apparatus

The computer hard- and software as well as the testing environment were identical to Experiment 1. The position of the right eye was recorded by means of a video-based Eyelink 1000 eye tracker (SR Research Ltd., Mississauga, Ontario, Canada), with a 1000-Hz temporal resolution, a 0.01° of visual angle spatial resolution (noise limited), and a gaze position accuracy of 0.5°.

#### Stimuli and procedure

The visual stimuli were identical to those used in Experiment 1 with the exception that instead of a black probe dot, the target stimulus was defined as the only right-tilted singleton among two left-tilted singletons and homogeneously oriented background lines. Moreover, in order to prevent target selection to be purely based on identity rather than salience information, the group of uniformly oriented background elements was either vertically or horizontally aligned. The sequence of events in a trial was identical to Experiment 1. Participants completed a total of 1080 experimental trials presented in random order, equally distributed across 54 blocks. Eye-movements were recalibrated 5 times during the experimental session upon completion of every 180 trials. On each trial, the right-tilted target stimulus was either the most, medium, or least salient element in the display. The orientation of the homogeneous background elements and the configuration of the three singletons were randomly varied across trials. Within a particular configuration, the locations of the most, the medium, and the least salient singleton were randomly assigned.

As in Experiment 1, prior to the testing session participants were presented with 3 practice blocks of 20 trials each, which were not included in any subsequent analysis. After completion of the testing session, participants were fully debriefed as to the purpose of the experiment.

#### Data analysis

The criteria used to extract fixation locations and durations of fixations and saccades were identical to those used previously.

The same analyses were performed on the data as described in Experiment 1. In addition, repeated measures analyses were run on the proportion of eye movements correctly directed towards the right-tilted target, averaged over all three salience conditions, with Response Time Bin (5) as independent within-subject factor. These analyses were run separately for initial and second saccades and the average performance was subsequently compared against chance level for each bin.

### Results and Discussion

Due to nonconformity to the previously established threshold criteria, 6.26% of initial saccades were excluded from analysis (2.70% due to an anticipation error (<80 ms), 0.70% due to the latency exceeding the threshold of 600 ms and 2.85% of initial saccades fell outside the range of 3 deg of visual angle of any of the three singletons.

#### Salience-driven influences on initial saccades

The procedure used to create the five bins for initial and second saccades was identical to the one used in Experiment 1.

**Figure 4:**
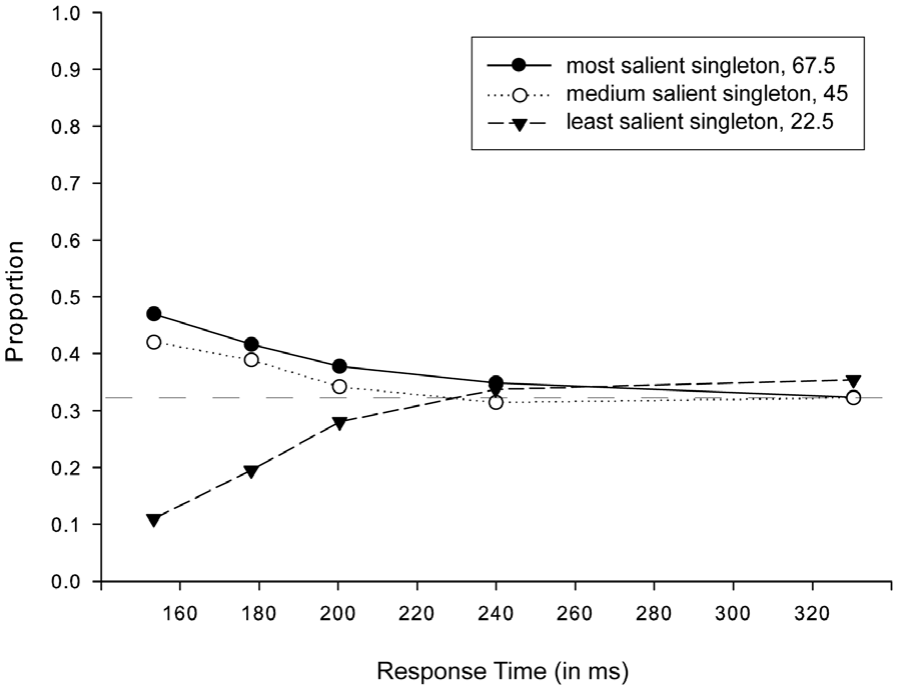
Salience-driven selection in initial saccades in Experiment 2. Proportions of initial saccades directed towards each of the three singletons (22.5°, 45°and 67.5°), separately for each bin of the response time distribution and irrespective of target identity.

The results of the ANOVA (see Figure 4) displayed a statistically significant main effect of Salience [F(1.104, 12.147) = 32.858, MSE = .016, η^2 = ^.315, p<.001], again qualified by a significant interaction between the Salience and Response Time Bin [F(8, 88) = 28.068, MSE = .007, η^2^ = .417, p<.001], indicating that the proportion of initial eye movements directed towards each of the three singletons varied as a function of response time. Post-hoc pair-wise comparisons between Salience separately for fastest and slowest responses revealed that while for fast-response saccades, participants were more likely to select a relatively high or medium salient singleton over a less salient singleton [t(11) = 9.514, p<.001 and t(11) = 10.792, p<.001, respectively], the proportion of eye movements directed to the most or medium salient singleton did not differ significantly [t(11) = 2.086, p = .061]. For the slowest-response saccades, participants were equally likely to make an eye movement to either of the three differently salient singletons (most versus medium salient: t(11)<1; most versus least salient: t(11) = -1.350, p = .204; and medium versus least salient: t(11) = -2.189, p = .051). In line with previous findings [14], [16], [17], and in particular Experiment 1, the results revealed that eye movements were primarily salience-driven for very fast responses (up to around 200 ms) after stimulus onset, whereas saccades elicited later in time were completely unaffected by salience.

#### Salience-driven influences on second saccades

For the analysis of second saccades only those trials were included in which the second eye movement landed on either of the two remaining singletons.

**Figure 5:**
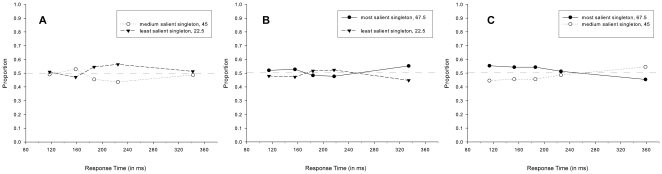
Salience-driven selection in second saccades in Experiment 2. Proportions of second saccades directed toward either of the two remaining singletons, separately for each bin of the response time distribution and irrespective of target identity, given that the initial saccade landed on A) the most, B) the medium, or C) the least salient singleton.

Three separate repeated measures ANOVAs (given the initial saccades landed on A) the most, B) the medium, or C) the least salient singleton) were performed on the proportions of second saccades directed toward either of the two remaining singletons with Salience (2) and Response Time Bin (5) as within-subject factors. The results of all three analyses (see Figure 5) revealed neither a significant main effect of Salience [for A): F(1, 11) = 3.117, MSE = .016, η^2^ = .057, p = .105, n.s.; for B): F(1, 11) = 1.329, MSE = .014, η^2^ = .025, p = .273, n.s.; and for C): F(1, 11) = 2.065, MSE = .028, η^2^ = .044, p = .179, n.s.], nor a significant interaction between Salience and Response Time Bin [for A): F(4, 44) = 2.591, MSE = .012, η^2^ = .142, p = .05, n.s.; for B): F(4, 44) = 2.354, MSE = .010, η^2^ = .134, p = .068, n.s.; and for C): F(4, 44) = 2.226, MSE = .018, η^2 = ^.122, p = .082, n.s.]. In line with Experiment 1, this suggests that salience-driven effects are limited to initial eye movements, and are completely irrelevant to visual search for eye movements elicited after approximately the first 200 ms after stimulus onset.

In addition to the time-course of salience-driven processes, another goal of Experiment 2 was to investigate whether goal-driven processes also develop according to the absolute time-course view. To this end, all following analyses were performed on accuracy.

#### Goal-driven influences on initial saccades

**Figure 6:**
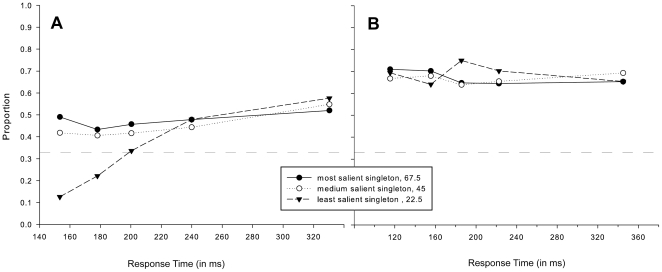
Goal-driven selection in Experiment 2. Proportions of eye movements correctly directed toward the right-tilted target singleton, averaged over all salience conditions, separately for each bin of the response time distribution for A) initial and B) second saccades.

A repeated measures ANOVA was performed on the proportions of initial saccades correctly directed towards the right-tilted singleton, averaged over all salience conditions, with Response Time Bin (5) as within-subject factor. The results (see Figure 6A) displayed a statistically significant main effect of Response Time Bin [F(1.814, 19.953) = 18.982, MSE = .010, η^2^ = .633, p<.001], attributable to an increasing proportion of initial eye movements correctly directed toward the target with increasing response time. In line with previous findings [17], this suggests that goal-driven processes unfold at a different rate than salience-driven processes. Indeed, compared with the pattern of salience-driven processes, the influence of goal-driven processes follows a reversed pattern, with visual search being unaffected by top-down control for very fast responses but primarily goal-driven for saccades elicited later in time.

#### Goal-driven influences on second saccades

A similar analysis was performed for the proportion of second saccades correctly directed towards the target. The results (see Figure 6B) of the repeated measures ANOVA revealed no significant main effect of Response Time Bin [F(4, 44) = .356, MSE = .003, η^2 = ^.028, p = .838, n.s.], indicating that performance in target selection did not vary with response time. Separate t-tests comparing each bin against chance level confirmed that performance accuracy differed significantly from chance [p<.001 for all bins] with performance accuracies around 70% across all bins. Thus, irrespective of response time and target salience, participants' performance was equally good at selecting the right-tilted singleton, indicating that visual search was primarily goal-driven during second saccades.

A major motivation for Experiment 2 was to decrease the response times of second saccades in order to increase the likelihood of finding salience-driven effects. In Experiment 2, the fastest saccades were initiated on average 100 ms earlier compared to those in Experiment 1, showing that the manipulation was successful. As salience effects are more likely to be found for fast-response saccades, the absence of an effect on fast responses of second saccades in Experiment 2 demonstrates that second eye movements are indeed completely unaffected by salience and not merely an inevitable result of the increased response times of second saccades as found in Experiment 1.

Comparing the findings of salience-driven and goal-driven processes obtained in Experiment 2, it is evident that the time-courses of both processes develop in a complementary fashion over multiple eye movements, in line with the absolute time-course view. For very fast responses of initial saccades, up to approximately 200–250 ms after stimulus onset, visual search was primarily salience-driven, whereas it was unaffected by goal-driven processes. With increasing response time, visual search became increasingly top-down controlled and less salience-driven. Crucially, this pattern was not found for second saccades, with visual search being continuously goal-driven and unaffected by salience-driven processes, irrespective of response time.

## Discussion

The goal of the current study was to investigate how salience-driven (Experiments 1 and 2) and goal-driven (Experiment 2) processes unfold during visual search over multiple eye movements (especially second eye movements), while taking into account the response time of each individual eye movement.

Regarding the contribution of salience-driven processes to the control of the initial saccades, the results of both Experiment 1 and 2 resemble previous findings reported by Donk and van Zoest (2008). In line with these findings, saccades elicited shortly after the onset of the search display were primarily salience-driven, whereas saccades that were elicited later in time, after approximately 200 ms, were completely unaffected by salience. More importantly, the results of both Experiment 1 and 2 unambiguously revealed that second saccades were completely unaffected by salience, irrespective of whether an eye movement was elicited early or late in time, that is, irrespective of response time. Together, these results suggest that the effect of stimulus-salience is crucially time-dependent, in line with the absolute time-course view, exclusively operating in an extremely brief time interval for approximately 200 ms after stimulus onset.

The contribution of goal-driven effects seems to develop in a complementary fashion, gradually building up over time. Indeed, the results of Experiment 2 revealed that eye movements elicited shortly after stimulus onset were completely unaffected by top-down control. However, for slower responses of the initial saccades, eye movements were increasingly guided by top-down control. Critically, search was completely goal-driven with consistently high performance across all response time bins of second saccades.

One might argue, however, that based on the paradigm it is not surprising to find an absence of salience effects on second saccades. That is, through removal of the fixation cross with stimulus onset, initial saccades might have been inevitably faster than second saccades, as the disengagement of attention from fixation has been exogenously pre-performed [39]. While response times of second saccades were indeed longer than those of initial saccades in Experiment 1, this was not the case in Experiment 2. In fact, response times of second saccades in Experiment 2 were not only around 100 ms faster than those of Experiment 1 but crucially even faster than the briefest responses of initial saccades. Thus, the additional step of disengagement of attention for second saccades does not seem to be inevitably associated with a cost in response time for those saccades. Given the speed of second saccades in Experiment 2, the circumstances were optimal for potential effects of salience to become evident; finding that stimulus salience did not affect these very fast second saccades strongly corroborates an absolute time-course view of salience-driven effects in visual search.

### Relation to findings of persistent effects of salience

How do these results relate to previous findings of salience-driven and goal-driven effects in overt visual search? While the findings of our present study are clearly consistent with studies indicating that visual search is primarily under top-down control, they seem to contradict findings showing persistent effects of salience over time [21]–[23]. This apparent discrepancy might arise from a fundamental difference in paradigms used across studies. Those studies finding persistent effects of salience usually employ images of complex natural and artificial scenes in a free-viewing paradigm, that are intrinsically susceptible to two potential limitations. Given that images depict objects, whether natural or artificial, and given that objects tend to contain the most salient regions in a display, it might be possible that the “persistent effects of salience” do not represent salience effects per se but rather object-presence effects. Indeed, recently Einhäuser, Spain, & Perona (2008) found that observers preferentially fixate “interesting” objects rather than salient regions in an image and concluded that salience only indirectly affected visual search, acting through recognized objects [40], [41]. In other words, salience is only effective if objects tend to be more salient than the background, but does not guide search directly. Given that salience is intricately linked with object presence, it is conceivable that the operationalization of salience in previous studies may reflect the potential impact of object presence rather than stimulus-salience [42].

Another potential limitation that is related to the fact that object-presence co-varies with salience, is the central fixation bias [21], [43]–[48]. As images are usually taken in a way that objects are located in the center of the image, salience effects might not only covary with object-presence effects, but observers might persistently fixate salient regions because they happen to be located in the center of the image. Due to the simple stimuli and the highly controlled paradigm used in the present study, any potential limitations due to central bias or object-presence effects are eliminated, lending unambiguous support to the absolute time-course view of salience-driven and goal-driven processes, assuming that visual search is primarily under top-down control following a brief period of initial salience-driven effects just after stimulus onset.

### Goal-driven control and trans-saccadic memory

It is evident from our results that the relative contribution of salience-driven and goal-driven processes obtained for initial saccades differs fundamentally from that obtained for second saccades. How can we account for this difference in eye movement behavior over multiple saccades? Taking into account findings from oculomotor research on trans-saccadic memory, initial saccades differ from all following saccades in the amount of information that is available concerning the stimulus display. Studies in this field have demonstrated that information that has been acquired from the visual periphery during one fixation is carried-over to the following saccade, thereby affecting the pattern of subsequent eye-movements [49]–[52]. Incorporating these findings into our results, we can account for the pattern of salience-driven and goal-driven processes over time as follows: Prior to the presentation of a stimulus display, no information regarding the properties of the stimuli is available. Therefore, the initial eye movement is purely driven by the relative salience of features if it is initiated within approximately 200–250 ms after presentation onset. Initial eye movements that are initiated past this crucial time interval are already, at least partly, guided by top-down control, as the more time passes between presentation onset and the initiation of a saccade, the more likely it becomes that information about stimulus properties is acquired from the visual periphery. This information accumulates over time, being carried over from one fixation to the next. Thus, every following eye-movement is primarily top-down controlled, drawing on information acquired over previous fixations. This suggests that the relative contribution of salience-driven and goal-driven processes to visual search is only indirectly time dependent, contingent upon the amount of information that is available prior to a saccade. This has implications for the stimuli used and can potentially account for differences found between studies using static and those using dynamic scenes. For static scenes, the amount of information available about a scene increases as a function of time. The scope of the present study forecloses any conclusion regarding the pattern of eye movements for dynamic scenes. It can be speculated that, under dynamic viewing conditions, the relative contribution of salience-driven and goal-driven processes over time operates differently, as the information contained in the scene is continuously changed. Whether this implies a larger contribution on the part of salience-driven processes, either through a larger continuous effect of salience or through salience being reinstated after every fixation remains an intriguing question for future research.
